# Relationship between commuting and health outcomes in a cross-sectional population survey in southern Sweden

**DOI:** 10.1186/1471-2458-11-834

**Published:** 2011-10-31

**Authors:** Erik Hansson, Kristoffer Mattisson, Jonas Björk, Per-Olof Östergren, Kristina Jakobsson

**Affiliations:** 1 Division of Occupational and Environmental Medicine, Faculty of Medicine, Lund University, Lund, Sweden; 2 Department of Occupational and Environmental Medicine, Regional University Laboratories, Lund, Sweden; 3 Competence Centre for Clinical Research, Skåne University Hospital, Lund, Sweden; 4 Social Medicine and Global Health, Faculty of Medicine, Lund University, Malmö, Sweden

## Abstract

**Background:**

The need for a mobile workforce inevitably means that the length of the total work day (working and traveling time) will increase, but the health effects of commuting have been surprisingly little studied apart from perceived stress and the benefits of physically active commuting.

**Methods:**

We used data from two cross-sectional population-based public health surveys performed in 2004 and 2008 in Scania, Sweden (56% response rate). The final study population was 21, 088 persons aged 18-65, working > 30 h/week. Duration (one-way) and mode of commuting were reported. The outcomes studied were perceived poor sleep quality, everyday stress, low vitality, mental health, self-reported health, and absence from work due to sickness during the past 12 months. Covariates indicating socioeconomic status and family situation, overtime, job strain and urban/rural residency were included in multivariate analyses. Subjects walking or cycling to work < 30 min were used as a reference category.

**Results:**

Monotonous relations were found between duration of public transport commuting and the health outcomes. For the category commuting > 60 min odds ratios (ORs) ranged from 1.2 - 1.6 for the different outcomes. For car commuting, the relationships were concave downward or flat, with increasing subjective health complaints up to 30-60 min (ORs ranging from 1.2 - 1.4), and lower ORs in the > 60 min category. A similar concave downward relationship was observed for sickness absence, regardless of mode of transport.

**Conclusions:**

The results of this study are concordant with the few earlier studies in the field, in that associations were found between commutation and negative health outcomes. This further demonstrates the need to consider the negative side-effects of commuting when discussing policies aimed at increasing the mobility of the workforce. Studies identifying population groups with increased susceptibility are warranted.

## Background

In current Swedish and European labour policy, increased mobility of the workforce is seen as an important way to create dynamic regions and thereby economical growth [1-3]. The average commuting time in the workforce in Sweden in 2000 was 37 minutes, placing Sweden just below the median when compared to the countries in the European Union and far below the US [4]. There are differences in the pattern of commuting between population groups in Sweden: young people, men, well-educated and persons born in Sweden more often commute to another local labour market than their contraries [2]. There are also differences in modes of commuting: women more often walk, cycle or use public transport to get to work than men [5].

Commuting has been shown to be associated with stress and fatigue, but also to be a possibility for recreation [6]. Perceived stress while, or immediately after commuting, has been found to increase with duration [7,8], variability in commuting time [9], lack of predictability [10], lack of control [8], and crowding [11]. Apart from increased physical activity and reduction in obesity [12], active commuting by walking or cycling is perceived as more relaxing and exciting than commuting by car or public transport, which is reported as being more stressful and boring, respectively [13].

Commuting has also been associated with negative health outcomes not directly related to the commuting situation itself. This may be mediated through the interdomain transfer effect (spill-over) of a negative commuting situation [14], or be the result of having less time for health-promoting behavior such as physical activity, relaxation and social participation. Short sleeping time has been observed among commuters in the US [15] and Italy [16]. In a previous Swedish study commuting was found to be weakly associated with being on full-time sick leave instead of part-time sick leave (during a period longer than 2 weeks) more frequently, and with increased sick leave of any type among low-paid women [17]. Increased absenteeism associated with commuting, especially among women, has been seen in Italy and elsewhere [9,16,18]. In the US, commuting has been shown to be associated with lower social participation [19-21], which has in turn been associated with health outcomes [22]. Commuting has been studied as one of the antecedents of work-family(/life)-conflict and is thereby related to lower general wellbeing and reduced physical and mental health [23,24].

Commuting has been considered a utility equilibrium with commuters being compensated for their efforts by higher salary, shorter working hours [4] and/or cheaper housing [25]. However, if one focuses on wellbeing instead of financial aspects as indicators of utility, this equilibrium theory is rejected by evidence of lower subjective wellbeing with longer commutes [4]. A useful framework for understanding this discrepancy between theory and empiricism has been presented by Ettema et. al. [26], who discusses the frequently observed divergence between decision utility (the utility that the individual expects before making a choice, in this case starting to commute), and experienced utility (the actual utility experienced when the decision has been made, i.e. when the person is actually commuting).

The aim of the present study is to further elucidate the association between commuting and health outcomes, using a large dataset that enables adjustment for several covariates in a multivariate analysis. We used cross-sectional data gathered from public health surveys to focus on the commuter's subjective perception of his or her general state of health. It was of special interest to investigate whether the effects of commuting were dependent on commuting mode. In the analysis we therefore considered commuting as a two-dimensional exposure with a quantitative (time) and a qualitative (mode) dimension.

## Methods

### Study area

**Figure 1:**
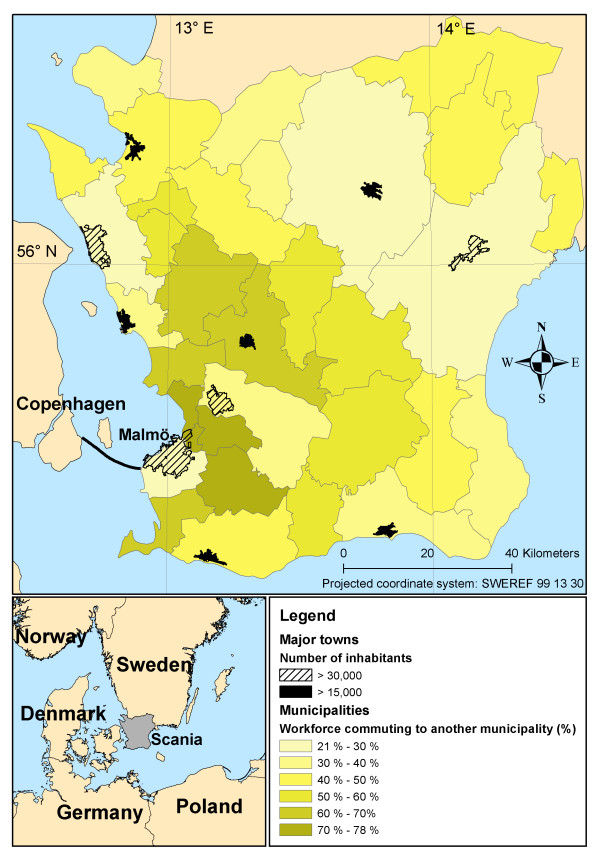
**Cross-municipality border commuting in study area**. Data from Statistics Sweden [27].

Scania (*Skåne*) is the southernmost county of Sweden (Figure 1), and with its 1.2 million inhabitants spread over 11,000 sq km, it is also one of the most densely populated counties (112 inhabitants/km^2 ^[27]). Both the population and job opportunities are concentrated to the west coast [28]. In 2005, 50% of the inhabitants lived in towns with more than 15,000 inhabitants, and four towns had more than 30,000 inhabitants, of which Malmö was the largest with 258,000 inhabitants [27]. There is a public transport system, administrated by the county council, that provides train and bus services in urban, suburban and, to a lesser extent, rural areas. There were 130 million trips made using the public transport system in Scania in 2009, of which 27% were trips by train [29]. The most common mode of commuting in the area is by car (71% of men, 56% of women) [28], which is a flexible, although expensive, alternative to public transport. There are relatively extensive networks of bike lanes throughout the region, encouraging cycling, and like most European regions the inner-city areas are comparatively well suited for walking. Commuting has increased substantially in Scania during the past decades [1]. The percentage of the workforce commuting over a municipality border tripled between 1970 and 2008 (from 13% to 39% [27,30]), and the number of train journeys has increased by ten times over the past 25 years [1]. Ten years after the construction of the Öresund bridge, connecting Malmö to the Danish capital Copenhagen, 19,000 Swedes commute to Denmark [31].

### Data gathering and selection criteria

Public health questionnaires had been sent out, by post, to a random sample of 99,763 inhabitants in Scania (47,621 in 2004, and 52,142 in 2008), aged 18 - 80 years. The random sample was weighted by age, sex and geographic area. A total of 56, 161 (27,963 in 2004, and 28,198 in 2008) persons responded, yielding a 56% response rate [12,32]. Men, young people, those with a low level of education and persons born outside Sweden responded to the questionnaire to a lower degree [32]. The two surveys from 2004 and 2008 had very similar content, and all questions included in the present study were identical. Here, we restricted the study group to respondents younger than 65 years (the standard retirement age in Sweden) who were working more than 30 hours per week, resulting in 23,111 subjects. Among these, 21,245 had responded to questions about commuting time and commuting mode. Subjects commuting only by walking or cycling for more than 30 minutes (one way) (167 subjects) were considered a too small group to be analyzed separately and were therefore excluded. Thus, the final study population was 21,088. The surveys were conducted by the Scania Regional Health Authorities, who made the final database available to us. As there was no possibility to identify respondents in this database, no approval from an ethics committee was required for this study.

To check for potential cluster effect, i.e. falsely narrow confidence interval due to correlation within the samples from the two survey years, a variable for survey year was included in the analysis. The estimations of the ORs of the exposure variable or their confidence intervals changed only marginally when survey year was added to the model. The OR estimates presented in the rest of the article are not adjusted for by survey year.

### Exposure variables

Commuting time was obtained in response to the question "How long time does it take to get to work (single journey)?", to which there were six different possible responses: 1) less than 15 minutes 2) 15 - 30 minutes 3) 30 - 60 minutes 4) 1 - 1, 5 hours 5) 1, 5 - 2 hours and 6) Longer than 2 hours. Commuting mode was determined by asking: "How do you usually get to work?". Those answering walking or cycling only were classified as Active commuters, those answering car but not bus or train were classified as Car commuters and those answering bus or train were classified as Public transport commuters. This coding was chosen as it gives priority to the more inflexible commuting mode, i.e. those with a combination trip that involves taking the car to the train station will be coded as using the train.

In the main analysis, the information about commuting time and mode was combined for the creation of one exposure variable. Those classified as Active, all of them commuting less than 30 min, were used as reference category and those classified as Car and Public respectively were divided into different exposure groups based on their one-way commuting time (aggregated to < 30 min, 30 - 60 min and > 60 min). Thus, the final exposure variable consisted of seven categories of combinations of mode and time (1 Active + 3 Car + 3 Public), enabling comparisons between different modes of commuting.

### Outcome variables

Among the several potential outcome variables included in the questionnaire, we chose to analyse those that indicated different aspects of reduced general state of health that could be directly related to commuting (i.e. without a long induction-latency period) and thus observable in a cross-sectional study.

*• Perceived sleep quality*: Respondents were asked: "Do you think you get enough sleep to feel rested?", and given the following alternatives: 1) Yes, in general, 2) Yes, but not often enough, and 3) No, never or almost never. The responses were dichotomised: 1 and 2 were coded into "Good perceived sleep quality", 3 was coded into "Poor perceived sleep quality". This strict cut-off value was chosen to identify as cases only those strongly perceiving poor sleep quality.

*• Everyday stress: *Respondents were asked: "Do you feel stressed in your everyday life?", and given the following alternatives: 1) Yes, often, 2) Yes, sometimes, and 3) No, (almost never). The responses were dichotomised: 1 into "Stress" and 2 and 3 into "No Stress". Similarly as for the sleep quality question, this strict definition of perceived stress was set to only include those strongly perceiving stress.

*• Exhaustion*: The SF-36 vitality scale was used to assess exhaustion [33]. The four items (How much of the time during the past 4 weeks; 1."did you feel full of pep?", 2."did you have a lot of energy?", 3."did you feel worn out?", and 4."did you feel tired?") were answered using a 6-point scale (1. "all the time", 2. "most of the time" 3. "a good bit of the time", 4. "some of the time", 5. "a little of the time" and 6. "none of the time". The points were recoded to be in the same direction (i.e. for questions 3 and 4 the reverse order was used) and then summed. Those with a sum of ≥16 (i.e. on average the first alternative on the negative half on the recoded 6-point scale)), were coded as "Exhausted". The cut-off value was chosen based on the cut-off value used in an earlier Swedish study finding changes in diurnal salivary cortisol secretion among those identified as exhausted in this way [34].

*• Mental health: *The GHQ12 instrument was used to assess mental health. If three or more of the 12 items were answered on the negative half on a 4-point graded scale, this denoted poor mental health (for questions see [22]). This scoring method (0-0-1-1) is the most frequently used, and the cut-off value of 3 or more points is the most commonly chosen cut-off value [35].

*• Self-rated health*: Respondents were asked: "How do you feel right now, physically and psychologically, considering your health and your well-being?" and asked to record their answer on a 7-point graded scale [36]. The responses were dichotomised, with answers below 5 considered low self-rated health. This cut-off value was chosen on theoretical grounds, it was assumed that commuting would not be associated with severely reduced well-being but rather the absence of feeling perfectly well.

*• Sickness absence: *The respondent was asked to state how many days he or she had been absent from work during the past year due to sickness. Due to concerns that this continous variable would be sensitive to the choice of cut-off value, 0, 5 and 15 days of sickness absence were used as cut-off values in a sensitivity analysis, similar to the intervals used in the public sector in Sweden as indicators of low sickness absence [37].

As expected, all outcomes were significantly inter-related (all p-values < 0.001, Pearson Correlations 0.095 - 0.480).

### Confounders

The covariates were chosen based on theoretical assumptions that they were associated with commuting and the outcomes and did not constitute mediators. In the statistical analysis, we chose to add covariates in two steps. The covariates entered in the first step (in tables "partly adjusted") represent fundamental demographic and socioeconomic dimensions, as described in the following paragraph.

The covariates included in the first step were *sex, age *(4 categories: 18 - 34, 35 - 44, 45 - 54 and 55 - 64 years), *education *(3 categories: 9 years or less, 10 - 12 years and 13 years or more), *place of birth *(2 categories: those born in Sweden and those born in any other country), and *occupational class *(job title and work tasks classified into 6 categories using the Swedish socioeconomic classification [38]: "unskilled" and "skilled" manual workers, non-manual employees on a "low", "medium" or "high" level, and "farmers and entrepreneurs").

In the second step, seven additional covariates were entered to the model together with the five added in the first step (in the tables, the estimates obtained from these analyses are termed "fully adjusted"). The seven covariates added in this second step comprises additional covariates with likely associations with both health outcomes and commuting; *job strain, overtime, history of unemployment, income, financial stress, residential location and family situation*.

Information about *residential location *was obtained through the sampling stratum information. Geographical strata in four towns/cities (Malmö (258,000 inhabitants), Helsingborg (91,000), Lund (76,000) and Kristianstad (33,000)) were coded as urban areas. Information about *family situation *was obtained through the question: "With whom do you share housing?" the response to which could be one or more of the following alternatives: Nobody, Parent/Sibling, Husband/Wife/Cohabiting/Partner, Other adult(s), and Children, which were divided into four age ranges: 0-6 years, 7-12 years, 13-17 years and 18 years and older. The answers were combined to give 4 categories combining form of cohabitation with having children under 13 (i.e. single-living with a child under 13, single-living without a child under 13, cohabiting and with a child under 13, and cohabiting and without a child under 13), and entered as a categorical variable.

Psychological demands and degree of control at work was assessed using JCQ (Job Content Questionnaire [39]). The cut-offs for high psychological demand and low degree of control were set to the median for each of the two survey years. Those classified as having both high psychological demands and low level of job control were classified as having *job strain. Overtime *was assessed through the question: "Do you work overtime?" with the alternatives: "I never work overtime", "I work overtime a few times per month", "I often work overtime" and "I do not have regulated working hours". Information about *history of unemployment *was obtained through the question: "Have you been involuntarily unemployed during the past three years?". Information about *income *was obtained from register data (SCB (Statistics Sweden)). The incomes for the two different years were adjusted for inflation by dividing the values for each year by an index value for that year, which is adjusted annually according to the consumer price index, and it was entered as a continuous variable.

As an indicator of *financial stress*, respondents were asked, "How often during the past 12 months have you had difficulties paying your bills (rent, electricity, telephone, mortgage, insurance, etc.)?", and given four alternatives: "Every month", "About half of the months", "A few times" and "Never". This was entered as a categorical variable in the regression models. In Table 1 those answering that they had difficulties paying bills "A few times" or more often were dichotomised into "Ever having problems paying bills".

**Table 1:** Descriptive data

	Total	Active < 30 min	Car < 30 min	Car 30 - 60 min	Car > 60 min	Public > 30 min	Public 30 - 60 min	Public > 60 min
Age^1 ^(median years)	45	46	45	45.5	47	43	43	43

Sex^1 ^(% male)	50	42	54	63	76	37	38	50

University education^1^ (%)	43	47	38	43	46	47	59	64

Manual workers^1 ^(%)	34	35	37	29	29	33	22	20

Born abroad^1 ^(%)	9	9	8	7	10	12	9	16

Urban residents^2 ^(%)	26	44	19	12	15	37	33	38

Cohabiting^2 ^(%)	78	72	80	83	82	72	77	75

Living with children under 13 years^2 ^(%)	31	24	34	33	31	30	29	25

High psycological demands at work^3 ^(%)	47	45	46	50	58	45	45	51

Low control at work^3^ (%)	53	55	53	46	38	59	57	53

Job strain^2 ^(%)	22	22	22	29	20	25	24	24

Frequently working overtime^2 ^(%)	25	24	26	28	37	19	21	27

Unemployed during the last three years^2 ^(%)	10	9	8	10	13	12	14	19

Income^2 ^(median number of price base amounts)	6.5	6.0	6.6	7.1	8.1	5.9	6.4	6.3

Ever having problems paying bills^2 ^(%)	22	20	22	21	23	26	24	26

Working hours per week^3 ^(median)	40	41	41	42	43	41	41	41

^1 ^Covariate adjusted for in the first step. ^2 ^Covariate adjusted for in second step. ^3^Covariate not adjusted for.

### Statistical analysis

Binary logistic regression modelling was used to determine possible associations between the commuting time/mode variable and each of the six health outcomes. Odds ratios with 95% confidence intervals (CI) were estimated from the models. After determining the unadjusted associations, the covariates were added in two steps as described above (results labeled "partly" and "fully adjusted" below and in Tables 2, 3, 4, 5, 6 and 7). PASW Statistics 18 was used for the calculations.

**Table 2:** Results of logistic regression analysis for the outcome *perceived poor sleep quality*

				Poor perceived sleep quality			
**Model**	**Commuting mode**	**Commuting time (min)**	**Resp.^1 ^(n)**	**Prev.^2 ^(%)**	**OR**	**95% C.I. Low**	**95% C.I. High**

Unadjusted	Active	< 30	4376	11	1.00		

	Car	< 30	10755	11	1.09	0.97	1.22

	Car	30-60	2280	13	1.30	1.12	1.52

	Car	> 60	449	10	0.94	0.68	1.30

	Public	< 30	1332	10	0.96	0.78	1.18

	Public	30-60	1312	13	1.27	1.05	1.53

	Public	> 60	584	15	1.55	1.21	1.97

Partly adjusted	Active	< 30			1.00		

	Car	< 30			1.09	0.97	1.22

	Car	30-60			1.37	1.17	1.61

	Car	> 60			1.05	0.76	1.46

	Public	< 30			0.91	0.74	1.11

	Public	30-60			1.24	1.03	1.50

	Public	> 60			1.53	1.19	1.96

Fully adjusted	Active	< 30			1.00		

	Car	< 30			1.05	0.93	1.19

	Car	30-60			1.37	1.16	1.62

	Car	> 60			1.02	0.72	1.45

	Public	< 30			0.85	0.69	1.05

	Public	30-60			1.17	0.95	1.43

	Public	> 60			1.41	1.08	1.85

1. Respondents; 2. Prevalence; Partly adjusted: Adjusted for the covariates *sex, age, education, place of birth *and *occupational class*; Fully adjusted: Adjusted for the covariates *sex, age, education, place of birth, occupational class, job strain, overtime, history of unemployment, income, financial stress, residential location *and *family situation*.

**Table 3:** Results of logistic regression analysis for the outcome *everyday stress*

				Everyday stress			
**Model**	**Commuting mode**	**Commuting time (min)**	**Resp.^1 ^(n)**	**Prev.^2 ^(%)**	**OR**	**95% C.I. Low**	**95% C.I. High**

Unadjusted	Active	< 30	4376	17	1.00		

	Car	< 30	10755	17	1.04	0.95	1.14

	Car	30-60	2280	18	1.11	0.97	1.26

	Car	> 60	449	16	0.98	0.75	1.27

	Public	< 30	1332	15	0.90	0.76	1.06

	Public	30-60	1312	20	1.24	1.06	1.45

	Public	> 60	584	20	1.25	1.00	1.55

Partly adjusted	Active	< 30			1.00		

	Car	< 30			1.10	1.00	1.22

	Car	30-60			1.25	1.10	1.43

	Car	> 60			1.21	0.93	1.58

	Public	< 30			0.84	0.71	0.99

	Public	30-60			1.18	1.00	1.38

	Public	> 60			1.23	0.98	1.53

Fully adjusted	Active	< 30			1.00		

	Car	< 30			1.11	1.00	1.24

	Car	30-60			1.28	1.10	1.49

	Car	> 60			1.11	0.83	1.49

	Public	< 30			0.81	0.67	0.98

	Public	30-60			1.19	1.00	1.42

	Public	> 60			1.19	0.93	1.53

1. Respondents; 2. Prevalence; Partly adjusted: Adjusted for the covariates *sex, age, education, place of birth *and *occupational class*; Fully adjusted: Adjusted for the covariates *sex, age, education, place of birth, occupational class, job strain, overtime, history of unemployment, income, financial stress, residential location *and *family situation*.

**Table 4:** Results of logistic regression analysis for the outcome *low mental health*

				Low mental health			
**Model**	**Commuting mode**	**Commuting time (min)**	**Resp.^1 ^(n)**	**Prev.^2 ^(%)**	**OR**	**95% C.I. Low**	**95% C.I. High**

Unadjusted	Active	< 30	4376	14	1.00		

	Car	< 30	10755	13	0.93	0.84	1.03

	Car	30-60	2280	13	0.91	0.78	1.06

	Car	> 60	449	12	0.88	0.65	1.18

	Public	< 30	1332	15	1.12	0.94	1.33

	Public	30-60	1312	15	1.08	0.91	1.29

	Public	> 60	584	18	1.36	1.08	1.72

Partly adjusted	Active	< 30			1.00		

	Car	< 30			0.98	0.88	1.08

	Car	30-60			1.00	0.86	1.17

	Car	> 60			1.04	0.77	1.41

	Public	< 30			1.04	0.87	1.24

	Public	30-60			1.00	0.83	1.19

	Public	> 60			1.27	1.01	1.61

Fully adjusted	Active	< 30			1.00		

	Car	< 30			1.00	0.90	1.13

	Car	30-60			1.01	0.86	1.20

	Car	> 60			0.98	0.70	1.36

	Public	< 30			0.96	0.79	1.15

	Public	30-60			0.95	0.78	1.15

	Public	> 60			1.21	0.94	1.56

1. Respondents; 2. Prevalence; Partly adjusted: Adjusted for the covariates *sex, age, education, place of birth *and *occupational class*; Fully adjusted: Adjusted for the covariates *sex, age, education, place of birth, occupational class, job strain, overtime, history of unemployment, income, financial stress, residential location *and *family situation*.

**Table 5:** Results of logistic regression analysis for the outcome *low self-rated health*

				Low self-rated health			
**Model**	**Commuting mode**	**Commuting time (min)**	**Resp.^1 ^(n)**	**Prev.^2 ^(%)**	**OR**	**95% C.I. Low**	**95% C.I. High**

Unadjusted	Active	< 30	4376	21	1.00		

	Car	< 30	10755	22	1.06	0.97	1.15

	Car	30-60	2280	23	1.08	0.95	1.22

	Car	> 60	449	22	1.04	0.82	1.31

	Public	< 30	1332	24	1.13	0.98	1.31

	Public	30-60	1312	23	1.07	0.92	1.24

	Public	> 60	584	27	1.35	1.10	1.64

Partly adjusted	Active	< 30			1.00		

	Car	< 30			1.08	0.99	1.17

	Car	30-60			1.19	1.05	1.34

	Car	> 60			1.22	0.96	1.55

	Public	< 30			1.09	0.94	1.27

	Public	30-60			1.10	0.94	1.28

	Public	> 60			1.48	1.20	1.81

Fully adjusted	Active	< 30			1.00		

	Car	< 30			1.12	1.02	1.23

	Car	30-60			1.25	1.09	1.43

	Car	> 60			1.20	0.92	1.56

	Public	< 30			1.05	0.89	1.23

	Public	30-60			1.06	0.91	1.25

	Public	> 60			1.44	1.16	1.80

1. Respondents; 2. Prevalence; Partly adjusted: Adjusted for the covariates *sex, age, education, place of birth *and *occupational class*; Fully adjusted: Adjusted for the covariates *sex, age, education, place of birth, occupational class, job strain, overtime, history of unemployment, income, financial stress, residential location *and *family situation*.

**Table 6:** Results of logistic regression analysis for the outcome low vitality

				Low vitaliy			
**Model**	**Commuting mode**	**Commuting time (min)**	**Resp.^1 ^(n)**	**Prev.^2 ^(%)**	**OR**	**95% C.I. Low**	**95% C.I. High**

Unadjusted	Active	< 30	4376	9	1.00		

	Car	< 30	10755	11	1.15	1.02	1.29

	Car	30-60	2280	11	1.16	0.98	1.37

	Car	> 60	449	9	0.91	0.65	1.28

	Public	< 30	1332	11	1.18	0.97	1.44

	Public	30-60	1312	12	1.27	1.04	1.55

	Public	> 60	584	13	1.47	1.13	1.91

Partly adjusted	Active	< 30			1.00		

	Car	< 30			1.21	1.07	1.36

	Car	30-60			1.38	1.17	1.64

	Car	> 60			1.19	0.84	1.69

	Public	< 30			1.11	0.91	1.36

	Public	30-60			1.34	1.10	1.64

	Public	> 60			1.64	1.26	2.14

Fully adjusted	Active	< 30			1.00		

	Car	< 30			1.22	1.07	1.39

	Car	30-60			1.42	1.18	1.71

	Car	> 60			1.26	0.87	1.82

	Public	< 30			1.05	0.85	1.30

	Public	30-60			1.30	1.05	1.61

	Public	> 60			1.55	1.17	2.07

1. Respondents; 2. Prevalence; Partly adjusted: Adjusted for the covariates *sex, age, education, place of birth *and *occupational class*; Fully adjusted: Adjusted for the covariates *sex, age, education, place of birth, occupational class, job strain, overtime, history of unemployment, income, financial stress, residential location *and *family situation*.

**Table 7:** Results of logistic regression analysis for the outcome *sickness absence > 5 days*

				Sickness absence > 5 days			
**Model**	**Commuting mode**	**Commuting time (min)**	**Resp.^1 ^(n)**	**Prev.^2 ^(%)**	**OR**	**95% C.I. Low**	**95% C.I. High**

Unadjusted	Active	< 30	4376	17	1.00		

	Car	< 30	10755	17	1.06	0.96	1.16

	Car	30-60	2280	17	1.03	0.90	1.18

	Car	> 60	449	13	0.75	0.56	1.00

	Public	< 30	1332	20	1.23	1.05	1.44

	Public	30-60	1312	19	1.15	0.98	1.34

	Public	> 60	584	16	0.94	0.74	1.19

Partly adjusted	Active	< 30			1.00		

	Car	< 30			1.11	1.00	1.22

	Car	30-60			1.20	1.04	1.38

	Car	> 60			0.94	0.70	1.26

	Public	< 30			1.20	1.03	1.42

	Public	30-60			1.23	1.05	1.45

	Public	> 60			1.08	0.84	1.37

Fully adjusted	Active	< 30			1.00		

	Car	< 30			1.15	1.04	1.27

	Car	30-60			1.27	1.10	1.47

	Car	> 60			1.03	0.75	1.41

	Public	< 30			1.14	0.97	1.35

	Public	30-60			1.16	0.98	1.38

	Public	> 60			0.96	0.74	1.25

1. Respondents; 2. Prevalence; Partly adjusted: Adjusted for the covariates *sex, age, education, place of birth *and *occupational class*; Fully adjusted: Adjusted for the covariates *sex, age, education, place of birth, occupational class, job strain, overtime, history of unemployment, income, financial stress, residential location *and *family situation*.

We tested the statistical interaction between commuting time and commuting mode by assessing the significance of departure from a multiplicative relation between commuting time and commuting mode on the odds ratio scale.

## Results

### Descriptive data

There were marked differences in commuting behaviour between demographic and socioeconomic groups in our study population (Table 1). Car and active commuters were generally older than those using public transport; more men than women used cars, and men commuted longer. Among those with long duration of commuting, especially by public transportation, a larger proportion had a university education. A higher percentage of the public transport commuters were immigrants. Car commuters lived in rural areas and had co-habitants and children to a greater extent. Job characteristics differed between commuting categories with *psychological demands, control, overtime *and *income *increasing with commuting time, and being higher for car commuters. The proportion that had been unemployed during the past three years increased with commute duration, and was higher among those using public transport. Public transport commuters more often experienced financial stress.

### Regression models

The health outcomes most clearly associated with commuting were *perceived poor sleep quality, exhaustion (low vitality) *and *low self-rated health*, whereas *low mental health *was not significantly associated with commuting (Tables 2, 3, 4, 5, 6 and 7). The association between commuting and *sickness absence *was sensitive to the choice of number of days of sickness absence during the past year used as cut-off value. For public transport users, ORs were increased with the 0 day cut-off (ORs 1.2 - 1.3, p < 0.05 in all commute duration categories) but was insignificant using the > 5 day cut-off (Table 7) and > 15 day cut-off. Car commuting was associated with *sickness absence *using the > 0 days cut-off (ORs at 1.1, p < 0.05 for time categories < 60 minutes) and also using > 5 days cut-off (Table 7) but insignificant using the > 15 days cut-off.

There was a close to statistically significant interaction between commuting mode and commuting time for two of the outcome variables (*perceived poor sleep quality*, p = 0.06 and *stress*, p = 0.08). The ORs for *perceived poor sleep quality *increased with commuting time among public transport commuters, whereas there was a curved association with time for car commuters (ORs peaking in the 30-60 min category). Interestingly, commuting for less than 30 minutes using public transport was significantly negatively associated with *stress*. For the other outcome variables interaction was less evident (*exhaustion*, p = 0.30; *low mental health*, p = 0.46, *low general self-rated health*, p = 0.20 and *sickness absence > 5 days*, p = 0.79).

The estimates of OR changed when adjusting for the two sets of covariates (Tables 2, 3, 4, 5, 6 and 7), especially when adjusting for the first set (*age, sex, education, country of birth *and *occupational class*). When these covariates were included in the model the estimates of OR for car commuting increased for all health outcomes, whereas the change in the estimates of the OR for public transport commuting were different for the various health outcomes.

Adding the second set of covariates (*job strain, overtime, history of unemployment, income, financial stress, residential location, family situation*) decreased the estimates of OR for public transport commuting for all outcomes, whereas there were mixed and mostly small changes in the estimates of OR for car commuting. Among the covariates included in the second step, the numerically most important were *financial stress *and *overtime*, which generally decreased the estimates, and *income*, which generally increased the estimates. However, their impacts on the ORs were generally modest.

The differences in choice of commuting mode related to sex, obvious in the descriptive data, raised concerns that the different patterns of association between mode of commuting and the outcomes were biased by gender. This hypothesis was tested by sex-stratifying the analysis of *perceived poor sleep quality*, which showed only minor sex-differences in the estimates (data not shown).

## Discussion

### Key results

The results of this study are in concordance with the findings of previous studies linking commuting to sleep disturbance [8,15,16], *everyday stress *[16], *exhaustion *[23], *low self-rated health *[4] and *sickness absence *[17]. Adjusting for confounding by demographic, socioeconomic, residential, work and family factors changed the strength of some of the associations, illustrating the complex relationships between commuting, health and other factors.

### Strengths and limitations

One strength of this study is the sample size, which enabled the analysis of associations between health and both the duration and mode of commuting. Moreover, the public health questionnaire had no specific focus on commuting and health, thus avoiding biased reporting. The data available allowed adjustment for several potential confounders, although there are other confounders that could not be adjusted for, such as shift work, commuting to Denmark and several contextual variables. Contextual variables that have not been addressed in this study include, for example, the availability of different types of work places, and the development of the public transportation system in the vicinity, which are not fully captured by the simple urban/rural dichotomy.

The overall response rate was 56%, and especially low among men, young people, those with a low level of education, low income and persons born outside Sweden [32]. This could mean that there is a lack of representativeness for these population groups, and that the findings of the study are more uncertain for these groups. No information about occupational status was available on individual level for the surveyed population, and we are therefore not able to do a formal analysis of the representativeness of the findings. Lack of time as a reason for not returning the questionnaire could constitute a source of bias in this study, as the commuters that perceive such a lack of time due to long commute duration might also be the ones whose health is also most affected. As this would lead to a lower response rate among individuals with the exposure, long duration commuting, and the outcome, e.g. poor self-rated health, this bias would reduce the observed association between commuting duration and health outcomes. Some of the subjects in this study might have been included twice. Unfortunately, there was no possibility of identifying these from the available data. Considering a population of 1 million and 50000 surveys in each year, 2500 persons might have received the survey twice. Assuming that those that responded in 2004 will respond also in 2008, and that those working > 30 hours/week in 2004 will do that also in 2008, and considering the overall response rate of 56% and inclusion rate of 38%, we would have approximately 500 (2%) duplicates in the dataset. Thus, we consider that a small proportion of subjects responding twice cannot affect the analysis importantly.

Since this is a cross-sectional study it is impossible to say that commuting caused the outcomes, and it is likely that other problems related to health and everyday life affect choices concerning commuting, leading to self-selection bias in our study. Less healthy people could be assumed to be less likely to start, or to continue commuting actively, creating bias away from the null when comparing active with passive transportation. An argument against why self-selection due to prevailing health problems is not the only cause of the association between the health outcomes and commuting is, however, that commuters using car or public transport for more than 30 minutes cover distances that could not be covered by walking or cycling for less than 30 minutes, i.e. there is little possibility to choose an active commuting mode for that distance. Another type of selection bias is also plausible, namely "the healthy commuter effect"; i.e. only those fit enough to endure commuting will start and continue long-distance commuting, whereas those experiencing lower utility or any type of health problems arising from commuting may choose to reduce their commuting time or change mode of commuting to minimize the impact of this strain on their life situation, creating bias towards null. The estimation of the commuting time might be affected by the mood of the respondent; those in a negative mood might be more likely to exaggerate the commute duration, i.e. dependent misclassification leading to increased estimates of association. A stressful life situation, in which it is perceived to be necessary to commute by car instead of the public transportation system [40], could be associated with negative health outcomes, meaning that there is reversed causation.

Having the financial security required for commuting long distances by car and the freedom to choose where to live, regardless of workplace location and public transport services, could also be associated with good health in a way that has not been completely adjusted for in our analysis. By considering the descriptive data, it is evident that long-duration car commuters are a relatively homogeneous and distinctive group, being male, well-paid and working overtime on jobs associated with high psychological demands and a high level of control. Another factor that should be considered is the availability of green environments close to one's home, which has been shown to be related to better general wellbeing [41] and vitality among women [42]. Green environments are more likely to be available in rural areas where long-distance car commuting is common.

The question about sleep might measure more of the subjective perception of the quality aspect of sleep than the objective quantity aspect. Thus this outcome variable might be more of a stress proxy than a measure of restricted time opportunities for sleep/recovery, which however is a potential consequence of everyday long-duration commuting. Both the questions concerning sleep and stress ask for subjective perceptions, and therefore there is nothing which can be objectively measured in order to estimate the validity of these questions. Several possible ways of coding SF-36 exist; we chose to use one which has been shown to be related to flattening of the diurnal cortisol profile in a sample of a working Swedes [34]. GHQ12 as a measurement of mental health is a short, but robust, measure of mental health [35]. The use of self-reported health in medical research is widespread, and poor self-reported health has been found to be associated with premature death [43]. Ettema et. al. [26] recently presented an extensive discussion on the use of subjective well-being as an outcome in transportation research. The 7-grade scale used in the present study has been found slightly more sensitive than alternative 5-grade scales [36].

The proportion of workers absent due to sickness less than 0, 5, 7 or 14 days per year is used as an indicator of a low sickness absence at workplaces in the public sector in Sweden [37].The analysis of the association between commuting and *sickness absence *was sensitive to the cut-off value used to classify high *sickness absence*. Different lengths of sickness absence in the previous year might reflect different types of negative health events, with long periods potentially caused by periods of actual disease, whereas few or short periods are more likely to also be influenced by "non-disease" factors. These "non-disease" factors could be commuting-related (such as the difficulties of getting to work when ill [18]), individual (e.g. the perception of need to stay at home when ill) and work-related (such as the possibilities to work while ill or work from home those days). This complexity could also be the explanation of the weakly decreasing trend we see for sickness absence with increasing commuting time: being generally more well-educated, long-distance commuters are more likely to have non-manual jobs that they partly can do from home, and thereby not have to be "absent" from work when feeling ill. Further research is necessary to elucidate the complex relationship between commuting and sickness absence, why the relationship between commuting and *sickness absence *presented in this study should only be seen as a preliminary estimate.

In a previous study of the association between commuting and utility it was argued incorrect to adjust for *overtime *and *income*, as these factors might constitute a compensation for commuting [4]. However, we believe that it is important to adjust for *overtime *in order to separate the association between commuting and health from other work-related factors or choices. *Income *is adjusted for since we consider the choice of commuting mode, where income could be a decisive factor. Another work-related factor we chose to adjust for is the psychosocial work environment (*job strain*) which can be expected to differ between short- and long-distance commuters. The current regulations concerning unemployment benefits in Sweden do not allow an unemployed person to restrict his or her job search geographically (until 2007, unemployed people were allowed to restrict their job search to the area/region in which they lived during the first 100 days of unemployment) [44] which could lead to longer commuting times among those with a *history of unemployment. Family situation *could constitute an important factor when choosing home and work location; for example, having children could motivate parents to commute in order to be able to raise their children in what they consider to be a good living environment [45]. However, parents, and perhaps especially single parents, would probably choose to avoid long commutes, if possible. *Financial stress *was included as financial problems could restrict the choice of work and home locations and mode of commuting. The *residential location *as conceptualised through the urban/rural dichotomy, is a factor that clearly affects commuting possibilities; those residing in an urban area have a wider selection of nearby workplaces than those living in rural areas, which could lead to generally shorter commutes in urban areas. Also, the availability of public transport is generally better in urban areas.

Although there are limitations to this study, we still consider it an important contribution to better understanding of the complex relationship between commuting and health. The study material is large, and we show associations with various health outcomes after adjustment for several covariates, congruent with findings from previous studies. Future research using more rigorous data gathering methods, and able to take into account additional, especially contextual covariates, could provide stronger evidence. Especially, longitudinal studies are needed.

### Interpretation

Although there was no significant departure from multiplicative interaction between commuting time and mode for any of the health outcomes, the suggested shapes of the associations between commuting time and the health outcomes were nevertheless different between public and car commuters.

The generally concave downwards association between car commuting time and health outcomes could be the result of the "healthy commuter effect" discussed above, or because those who need to use their car to commute short distances have poor health or are facing stressful life situation [40] in comparison to those being able to choose active commuting or public transport. We should also consider the possibility that long-distance car commuting may not actually be particularly harmful to health, especially in this geographical area, where a > 1-hour car commute does not imply > 1 hour of intense rush-hour traffic, but in most cases some tranquil countryside driving, which may offer the possibility of relaxation and give a feeling of flow [9]. Indeed, commuting a shorter distance by car may involve just as much time in stressful car driving, or even more than long-distance commutes, as these shorter journeys may be completely within urban or suburban areas.

A potential explanation of the generally more linearly dose-dependent association between commuting time and health outcomes among public transport commuters is that longer commuting times on public transport imply changes between buses or trains, and thus a higher risk of unpredictable and uncontrollable delays when commuting, which are potential causes of commuting-associated stress [9,10]. Long-distance commuting using public transport system could mean having to adjust one's everyday life according to bus or train timetables [1,46], which results in inflexibility and loss of control, with potentially negative effects on health.

Thus, fundamental demographic and socioeconomic factors lead to confounding of the relationships between commuting and health, and it is necessary to handle this complexity when studying such associations in future studies. We investigated whether additional, more specific factors, related to work, home and family conditions, would act as confounders, and included these in additional models. This generally lowered the ORs between commuting and health outcomes, especially for public transport commuting, although only slightly.

### Generalizability

Previous studies in Western countries have found associations between commuting and negative health outcomes [4,8,15-17,23,24], and the findings of our study are concordant with these findings. However, the specific associations between commuting time and health outcomes for different commuting modes are context dependent. The degree of traffic congestion, and the development of the public transportation network and bike lanes are only a few contextual factors that must to be considered when comparing the associations between commuting time and mode, and health in different geographical areas.

## Conclusions

The negative effects of commuting on health must be included in discussions on the expansion of economic regions and increasing the mobility of the workforce; a discussion that has hitherto been dominated by positive attitudes [1,46]. Research enabling us to clarify if and how commuting *causes *ill health would provide important information to help balance policies that lead to an increase in commuting, and in the development of measures to reduce the negative effects of commuting. Furthermore, studies to identify if any population groups are more likely to suffer from ill health because of commuting are warranted.

## Competing interests

The authors declare that they have no competing interests.

## Authors' contributions

EH drafted and wrote the main part of the manuscript and conducted the statistical analyses. KJ conceived the study, and JB, POÖ and KJ provided ideas for the analysis. POÖ lead the team which developed the original questionnaire and coordinated the data collection of the 2004 survey. All authors helped draft the manuscript, and read and approved the final manuscript.

## Pre-publication history

The pre-publication history for this paper can be accessed here:

http://www.biomedcentral.com/1471-2458/11/834/prepub
